# Economic Evaluations of Treatments for Inflammatory Bowel Diseases: A Literature Review

**DOI:** 10.1155/2018/7439730

**Published:** 2018-06-13

**Authors:** Lachaine Jean, Miron Audrey, Catherine Beauchemin, on behalf of the iGenoMed Consortium

**Affiliations:** ^1^Faculty of Pharmacy, University of Montreal, Montreal, QC, Canada; ^2^Montreal Heart Institute, 5000 Bélanger, Room S-6300, Montreal, QC, Canada H1T 1C8

## Abstract

**Objective:**

The objective of this literature review was to evaluate the existing evidence regarding the cost-effectiveness of treatment options in IBD.

**Methods:**

A systematic review of the literature was conducted to identify economic evaluations of IBD therapy. The literature search was performed using electronic databases MEDLINE and EMBASE. Searches were limited to full economic evaluations published in English or French between 2004 and 2016.

**Results:**

A total of 5,403 potentially relevant studies were identified. After screening titles and abstracts, 48 studies were included, according to the eligibility criteria. A total of 56% and 42% of the studies were assessing treatments of UC or CD, respectively. Treatment options under evaluation included biological agents, mesalamine, immunosuppressants, and surgery. The majority of studies evaluated the cost-effectiveness of biological treatments. Biological therapies were dominant in 23% of the analyses and were cost-effective according to a $CAD50,000/QALY and $CAD100,000/QALY threshold in 41% and 62% of the analyses, respectively.

**Conclusion:**

This literature review provided a comprehensive overview of the economic evaluations for the different treatment options for IBD over the past 12 years and represents a helpful reference for future economic evaluations.

## 1. Introduction

Inflammatory bowel diseases (IBD) are chronic, progressive, and disabling inflammatory conditions that affect the gastrointestinal (GI) track. Although not fatal, these conditions are associated with many symptoms, which have a major impact on patients' quality of life, including abdominal pain, fever, vomiting, diarrhea, rectal bleeding, anemia, and weight loss [1–3]. Patients with IBD often experience periods of remission alternating with periods of disease activity defined as relapse episodes [4, 5].

IBD consist primarily of Crohn's disease (CD) and ulcerative colitis (UC), which are distinguished by the location and the nature of the inflammation. Specifically, CD occurs most commonly in the ileum and colon although it can affect any part of the digestive system. This condition is associated with deep and transmural mucosal inflammation and is characterized by segmental inflammation along the GI track. Patients diagnosed with CD often suffer from fistulas and perianal impairments. As opposed to CD, UC is mostly associated with continuous and diffused inflammation. Thus, the inflammation is often limited to the inner lining of the colon and rectum area; patients with UC generally present symptoms such as bloody diarrhea as well as mucus or pus in stools.

As opposed to several other chronic or inflammatory diseases, IBD affect a young population, as the first onset is generally seen in early adulthood or even in late adolescence [6]. Several risk factors have been attributed to the onset of IBD, including the environment and Western lifestyle, which is associated with smoking, a diet rich in fat and sugar, excessive consumption of drugs, and high socioeconomic status. Genetic factors play an important role in disease susceptibility, with over 200 genetic loci being associated with CD and UC. Moreover, environmental factors, such as the intestinal flora, play a central role in the initiation and maintenance of disease [7]. In addition, immune factors, appendectomy, and stress may also affect the development of IBD [8].

Worldwide, Canada is among the countries with the highest IBD prevalence and incidence rates [9, 10]. It was estimated that, in 2012, there were 129,000 people living with CD in Canada, while over 5,700 new cases were diagnosed every year. A similar epidemiology pattern is seen in UC. In Canada, it was estimated that, in 2012, there were 104,000 people living with UC, while there were 4,500 new cases diagnosed every year [11]. The total prevalence of IBD in Canada is estimated at 1 in 150 Canadians (0.67% of the population). As opposed to Canada, incidence rates for CD and UC in Europe, between 1991 and 1993, were 7.0 and 11.8 cases per 100,000-person year, respectively [9]. Similarly, in the US, the reported incidence rates, between 1996 and 2002, for CD and UC were 6.3 and 12.0, respectively [10]. In 2004, more than 1.4 million residents in the US and 2.2 million in Europe suffered from IBD [9].

The main goal of current treatments for IBD is not to cure the disease, but rather to improve patients' quality of life and to decrease morbidity by inducing and maintaining remission [12, 13]. More precisely, for CD, conventional therapies are given as first-line treatments and comprise anti-inflammatory drugs such as glucocorticoids and mesalamine (aminosalicylic acid (5-ASA)) and immunosuppressive therapies such as 6-mercaptopurine (6MP), azathioprine (AZA), cyclosporine, or methotrexate (MTX) [14]. If a patient still has symptoms after first-line treatment with conventional therapy or is unable to tolerate conventional therapy, biological treatments could be considered as subsequent-line therapies. Biological therapies include anti-TNFs, such as infliximab (IFX), adalimumab (ADA), golimumab (GOL), and ustekinumab (UTK), and anti-integrin treatments, such as vedolizumab (VED). In the context of UC, 5-ASA is the cornerstone of the treatment of mild to moderate UC [15]. For moderate to severe UC, treatment should be initiated with corticosteroids, followed by 5-ASA, immunosuppressant, or biological agents. If the patient reaches clinical response, the therapy can be maintained. Otherwise, a biological agent in combination with immunosuppressive drugs can be administered [16]. Finally, for both CD and UC, surgeries, including colectomy (removal of the large intestine and rectum) and ileostomy (connecting the small bowel to an exterior bag), represent other treatment options [17].

The economic burden of IBD is substantial considering the prevalence of the disease and the high cost of treatment options. In 2012, Rocchi et al. conducted a literature review to establish the economic and epidemiological profile of IBD in Canada [18]. The authors evaluated the total annual cost to $CAD2.8 billion in 2012, which corresponds to approximately $CAD12,000 per patient affected by UC or CD. They estimated that the direct medical costs associated with IBD exceeded $CAD1.2 billion a year and were mainly attributable to drug costs ($CAD521 million), hospitalizations ($CAD345 million), and medical visits ($CAD132 million). Indirect costs totalized $CAD1.6 billion and were attributable mainly to the long-term productivity losses ($CAD979 million).

## 2. Objective

As the economic burden of IBD is significant, numerous economic evaluations assessing the cost-effectiveness of treatment options in IBD have been performed during the past years. The objective of this literature review was to explore the existing evidence regarding the cost-effectiveness of these treatments.

## 3. Methods

### 3.1. Literature Search

A systematic review of the literature was conducted to identify complete economic evaluations of IBD therapy. The review question was established using the PICO method [67]: population consisted of patients with IBD; interventions and comparators were standard therapies for IBD (drugs or surgery); outcomes of interest were results of cost-utility analyses (CUA), cost-effectiveness analyses (CEA), cost-minimization analyses (CMA), cost-consequence analyses (CCA), or cost-benefit analyses (CBA). CUA were expressed in terms of cost per QALY whereas CEA were expressed in terms of cost per remission, cost per response, cost per life year gained (LYG), cost per mucosal healing (MH), or cost per days without symptoms or steroids (DWSS).

A structured literature search was performed using electronic databases MEDLINE and EMBASE, in addition to a manual search of health technology reports and NICE technology appraisals that were not published in a peer-reviewed journal. PubMed was also searched to ensure that more recent studies (June 18th 2015 to June 18th 2016) not yet indexed in MEDLINE were identified. The keywords used for search were* “crohn disease”*,* “crohn's disease”*,* “ulcerative colitis”*,* “inflammatory bowel disease”, *and* “IBD”, *combined with the National Health Service Economic Evaluation Database (NHS EED) filters for economic evaluations. The search was limited to studies that were published in English or French, between 2004 and 2016 (June 18th). Furthermore, a cross-reference search was performed to identify additional publications.

### 3.2. Study Selection

Studies were initially selected based on titles and abstracts. Full-text articles of studies deemed eligible according to the abstract were then reviewed using a predefined eligibility form. Only full economic evaluations of IBD therapy available as full-text articles were included in this review. Studies were excluded if they were not full economic evaluations such as cost of illness, costs studies, or systematic review. All eligibility criteria were defined a priori. Study selection was performed by two independent reviewers for validation purposes. Disagreement between the reviewers was discussed and resolved by consensus.

### 3.3. Data Extraction

For each economic evaluation selected for inclusion, the following characteristics and parameters were extracted using a self-developed data extraction form: first author, journal, year of publication, title, type of funding, country, type of evaluation, time horizon, perspective, population, treatments of interest, comparators, type of model, cost description, outcome measures, utility values (including health states, source of utility values, methodology, and number of patients) and study results. Two reviewers independently extracted data to ensure appropriate validation.

### 3.4. Data Analysis

In order to provide a comprehensive overview of the economic evaluations of treatments for IBD, study characteristics were first summarized using descriptive statistics. In addition, characteristics, parameters, and results of economic evaluations for both UC and CD and for the different treatment options were assessed and compared. For comparison purposes, all the costs in this study have been transposed to 2016 Canadian dollars ($CAD) using the health and personal care component of the Canadian Consumer Price Index [68].

## 4. Results

### 4.1. Literature Search

**Figure 1:**
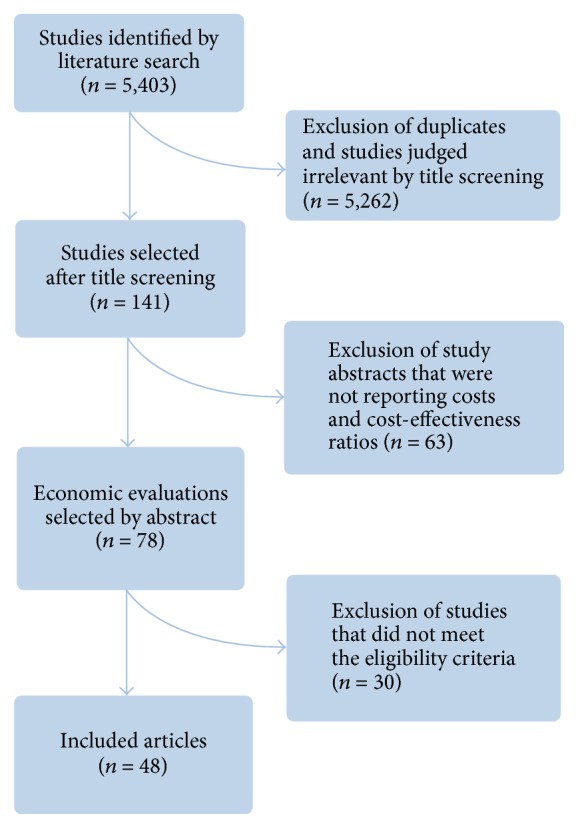
Study flowchart.

A flowchart describing the selection of studies included in this systematic review is presented in Figure 1. A total of 5,403 potentially relevant studies were identified by the literature search. After the screening of titles and abstracts, 78 full-text articles were assessed according to the eligibility criteria. Of these studies, 48 articles meeting inclusion criteria were included.

### 4.2. Overview of Included Studies

More than half of the included studies (56%) were assessing treatments of UC, 42% were evaluating treatments of CD, and only 1 study analyzed treatment for both CD and UC patients (Table 1). Different treatment options were evaluated such as IFX, ADA, other biological treatments (golimumab, natalizumab, or VED), 5-ASA, immunosuppressants (AZA, 6MP, MTX, or cyclosporine), surgery (colectomy or Ileal Pouch-Anal Anastomosis (IPAA)), and granulocyte-monocyte aphaeresis (GMA). Overall, most studies were CUA that assessed the cost-effectiveness of a new biological therapy from a healthcare system perspective over a time horizon of 1 year or less. Moreover, nearly half of the studies used standard of care as a comparator. Furthermore, Canadian, American, and European studies accounted for 13%, 29%, and 52% of all the studies, respectively.

### 4.3. Modelling Approach and Health State Definition

Half of the included studies used Markov modelling while one-third used decisions tree models. As for the remaining studies, some had no specific models employed [29, 30, 34, 65] or reported [33, 45]. The economic evaluations (EE) using a nonmodelling approach derived mainly from prospective data analysis or randomized clinical trials, whereas the studies not reporting their modelling approach were EE reports from the Canadian Agency of Drug Technology in Health (CADTH). Various criteria and scales, often based on previous clinical trials' remission and response definitions, defined models' health states. Among the studies assessing treatments for CD, a high proportion of the studies defined their health states according to Crohn's Disease Activity Index (CDAI), while some studies used the Harvey-Bradshaw Index (HBI). In economic evaluations assessing treatments for UC, most studies defined their health states according to the Mayo score, while others based their health states on symptom recurrence, on the Ulcerative Colitis Disease Activity Index (UCDAI) definition, on the Simple Clinical Colitis Activity Index (SCAI), or on the Physician Global Assessment (PGA).

### 4.4. Cost Parameters

Several cost parameters were taken into account in the included studies. More specifically, all studies reported drug costs, including curative and supportive treatment costs. Moreover, costs associated with hospitalization and outpatient visits were comprised in 73% and 69% of the studies, respectively, while costs associated with surgical procedures were reported in 67% of the studies. Imaging, lab tests (tuberculin skin, hepatitis B blood tests, and biochemistry testing), and endoscopy costs were included in 42%, 38%, and 17% of the study, respectively. Among studies assessing the cost-effectiveness of a biological treatment, 70% included infusion costs.

### 4.5. Outcomes

As most included studies were CUA, results were most frequently expressed in terms of cost per QALY. As for the CEAs, results were reported in terms of cost per response or remission, cost per DWSS, cost per LYG, and cost per MH.

### 4.6. Cost-Effectiveness Results

For comparison purposes, only CUA results for CD and UC were taken into account, which are presented in Tables 2 and 3, respectively.

#### 4.6.1. Cost-Effectiveness of Biological Therapies

The majority of studies evaluated the cost-effectiveness of biological treatments. More specifically, regardless of the IBD type and the comparative treatment, biological therapies were dominant in 23% of the analyses and were cost-effective according to a $CAD50,000/QALY and $CAD100,000/QALY threshold in 41% and 62% of the analyses, respectively. Biological treatments tended to be more cost-effective when compared with surgery (dominant in 43% and cost-effective according to a $CAD50,000/QALY ratio in 57% of the analyses) and when compared with other biological treatments (dominant in 48% and cost-effective according to a $CAD50,000/QALY ratio in 52% of the analyses), rather than with standard of care (dominant in only 8% of the analyses and cost-effective in 33% of the analyses according to a $CAD50,000/QALY threshold).

In CD, the incremental cost-effectiveness ratio (ICER) of biological treatments ranged from dominant to $CAD32,088,410/QALY when IFX or ADA maintenance treatment was compared to IFX or ADA induction treatment. Moreover, ADA tended to lead to more favourable ICERs than IFX when compared to standard of care, while IFX led to more favourable ICERs than ADA when compared to other biological treatments. Notably, moderate CD treatment regimens encountered greater cost-effectiveness ratios (CERs) compared to severe CD.

In the context of UC, dominance was mostly reported in studies where biological treatments were compared with other biological treatments or surgery. Moreover, all analyses were under a $CAD100,000/QALY threshold when IFX or ADA alone was compared with standard of care.

As for the Canadian setting, all studies in CD comparing biological treatments to standard (STD) of care resulted in an ICER above the $CAD100,000/QALY threshold. The opposite is seen in UC, where all studies resulted in an ICER under the $CAD100,000/QALY threshold. Furthermore, all ICERs resulting from the comparison of GOL to ADA were dominant.

#### 4.6.2. Cost-Effectiveness of Immunosuppressants

Most included studies assessing the cost-effectiveness of an immunosuppressant demonstrated that these treatments are cost-effective. More specifically, in the context of CD, AZA and cyclosporine were dominant alternatives when compared to therapy excluding immunosuppressants, when compared with MTX and when compared to standard of care. Moreover, cyclosporine was cost-effective according to a $CAD50,000/QALY threshold when compared with surgery.

In UC, only 1 economic evaluation was retrieved and indicated that immunosuppressant was a dominant alternative when compared to standard of care and was cost-effective according to a $CAD50,000/QALY willingness to pay threshold when compared with surgery.

#### 4.6.3. Cost-Effectiveness of Mesalamine (5-ASA)

All studies assessing the cost-effectiveness of 5-ASA were performed in the context of UC and compared 5-ASA with different 5-ASA formulations, doses, and treatment regimen. 5-ASA was dominant in 72.7% of the analysis and cost-effective according to a $CAD50,000/QALY willingness to pay (WTP) threshold in 81.9% of the analyses.

#### 4.6.4. Cost-Effectiveness of Surgery

Surgery was evaluated in treatments for UC only and was dominant when colectomy was compared with standard of care. However, when colectomy was performed at an early stage and combined with IPAA, surgery was not cost-effective according to a $CAD100,000/QALY WTP threshold compared to standard of care.

## 5. Discussion

Recent years have witnessed a rapid growth in IBD treatments. More specifically, the addition of biological treatments in the therapeutic arsenal of IBD has allowed significant clinical benefits, although it is associated with a substantial economic burden. Numerous economic evaluations have been performed in the last years in order to evaluate the cost-effectiveness of IBD treatments. The objective of this literature review was to explore the existing evidence regarding the cost-effectiveness of IBD treatments. This review found that a high proportion of biological therapies were cost-effective according to a $CAD100,000/QALY. Studies evaluating biological treatments in patients with severe disease and inadequate response to conventional therapies were found to be particularly cost-effective. Immunosuppressants and 5-ASA were also cost-effective strategies. On the other hand, the ranged ICER presented for IFX and ADA maintenance therapy versus induction therapy in CD was substantially wide. This variation could be explained by change in treatment regimen costs, despite the similarity between the associated QALY values. For the Canadian studies, the results seemed to differ by type of IBD, where ICERs for CD were much higher than ICERs for UC.

Up to now, other literature reviews on economic evaluations of IBD treatments were performed. Most of these studies assessed the cost-effectiveness of biological treatments only [24, 69–76], while only a few have taken into consideration all treatments for IBD [77, 78]. The present study is considering all IBD treatment options, including biological agents (IFX, ADA, GOL, NAT, and VED), immunosuppressants (AZA, 6MP, and cyclosporine), 5-ASA, GMA, and surgery (colectomy, IPAA). The findings of the present study are in line with the results of the previous literature reviews.

This study provides an exhaustive and complete overview of the economic evaluations performed in the context of IBD during the past years. A rigorous systematic review was conducted according to a predefined protocol, based on best practice guidelines. Even if this was not a specific selection criterion, economic evaluations included in this review were, in general, of good quality. Moreover, a 12-year time period was covered, which allowed the identification and the selection of a large number of relevant cost-effectiveness and cost-utility analyses. Such a long timeframe provided a good overview of the key characteristics of pharmacoeconomic analyses conducted in IBD during the last years. Furthermore, a high proportion of studies were of Canadian and American origin, which is in line with the high prevalence and incidence rates of IBD in theses' respective countries. Among other things, Canada detains one of the highest IBD prevalence and incidence worldwide.

However, this review has some limitations. This review was limited to English and French articles only. In addition, this review did not use a standardized tool for assessing the methodological quality of included studies. Another limitation involves the heterogeneity and variability of the characteristics and parameters of the studies included in this literature review. For instance, the methods used to assess the effectiveness differed from one study to the other. Many studies in UC or in CD used different disease progression index scores for definition of their model health states, including CDAI, HBI, or UCDAI scores. However, the latter scores are not based on the same patient disease characteristics and could therefore explain the variability in effectiveness among the studies. Moreover, different time horizons were chosen among included studies, which could have accounted for variability among studies. As IBD are chronic diseases, a longer time horizon allows better capturing remission and relapsing cycles and complications. However, a low proportion of studies have accounted for such a long time horizon. IBD complications, such as gastrointestinal cancers, are widely acknowledged as a long-term complication, likely as a result of chronic inflammation [79, 80]. Though, only few authors have incorporated colorectal cancer (CRC) risk in their economic model. In addition, the study population varied in terms of patients' age (adults or paediatric patients), previous exposition to treatment options (biological naïve patients, steroid refractory patients, and patients with inadequate response or medical contraindications for conventional therapies), and disease severity (patients with mild disease, patients with moderate to severe disease, patients with active luminal or fistulizing disease, and patients with acute exacerbations of disease).

Furthermore, the majority of selected economic evaluations have focused on a public healthcare system perspective, whereas only one study considered the societal perspective and led to a more favourable ICER than other studies comparing the same treatment options. IBD is a disease diagnosed as early adults, hence leading to a substantial impact on productivity loss and related costs. It has been demonstrated that biological therapies were associated with improved health outcomes, such as reduction in absenteeism [34, 81]. Considering that productivity losses account for a significant portion of the disease burden, the societal perspective is relevant and could have been considered [82]. Nevertheless, despite these limitations, this review adds to the current literature by providing a comprehensive overview of the existing economic evaluations in IBD therapy.

## 6. Conclusion

This literature review provided a comprehensive overview of the economic evaluations for the different treatment options for IBD over the past 12 years and represents a helpful reference for future economic evaluations.

## Figures and Tables

**Table 1:** Study Characteristics.

	Number of studies *n* (%)
*Type of analysis*	*n* (%)
CUA** **	40 (83)
CEA** **	5 (10)
CMA	3 (6)
*Study population*	
UC** **	27 (56)
CD** **	20 (42)
Both CD and UC	1 (2)
*Type of treatments under investigation*	
Biologic treatments** **	33 (69)
5-ASA** **	11 (23)
Immunosuppressant	5 (10)
Surgery	2 (4)
GMA	1 (2)
*Study perspective*	
Healthcare system perspective** **	47 (98)
Societal	1 (2)
*Time horizon*	
≤1 year** **	24 (50)
2–5 years** **	11 (23)
6–10 years** **	4 (8)
30 years** **	2 (4)
Lifetime** **	4 (8)
Not reported	3 (6)
*Model structure*	
Markov model** **	24 (50)
Decision tree** **	16 (33)
Markov and decision tree** **	2 (4)
No model	4 (8)
Not reported	2 (4)

CUA: cost-utility analysis; CEA: cost-effectiveness analyses; CMA: cost-minimisation analysis; GMA: granulocyte-monocyte aphaeresis; UC: ulcerative colitis; CD: Crohn's disease; 5-ASA: 5-aminosalicylic acid.

**Table 2:** Summary of economic evaluations in the treatment of CD.

Study, year of publication	Study treatment	Comparators	CE ratio (Cost/QALY) (Currency year)	CE ratio ($/QALY) (CAD 2016)
*IFX*				

Ananthakrishnan et al., 2011 [19]	IFX (tailored)	Antibiotics	Dominated	Dominated
	IFX (upfront)	Antibiotics	$2,757,857/QALY (USD 2010)	$3,121,546/QALY

Ananthakrishnan et al., 2013 [20]	IFX (MTN or dose escalation)	IFX (dose escalation)	$49,278/QALY (USD 2010)	$55,776/QALY

*Blackhouse et al., 2012 [21]*	*IFX*	*Std care*	*$222,955/QALY* *(CAD 2011)*^*∗*^	*$229,197/QALY*

Bodger et al., 2009 [22]	IFX (1 year tx)	Std care	£19,050/QALY (GBP 2006)	$49,290/QALY
	IFX (2 year tx)	Std care	£21,300/QALY (GBP 2006)	$55,112/QALY

Doherty et al., 2012 [23]	IFX	AZA/6MP	$1,831,912/QALY (USD 2010)	$2,073,493/QALY

Dretzke et al., 2011 [24]	IFX IND (severe disease)	Std care	Dominant	Dominant
	IFX MTN (severe disease)	Std care	£68,315/QALY (GBP 2011)^*∗*^	$157,706/QALY
	IFX MTN (severe disease)	IFX IND (severe disease)	£5,030,000/QALY (GBP 2011)^*∗*^	$11,611,849/QALY
	IFX IND (moderate disease)	Std care	£94,321/QALY (GBP 2011)^*∗*^	$217,741/QALY
	IFX MTN (moderate disease)	Std care	£317,991/QALY (GBP 2011)^*∗*^	$734,088/QALY
	IFX MTN (moderate disease)	IFX IND (moderate disease)	£13,900,000/QALY (GBP 2011)^*∗*^	$32,088,410/QALY

Jaisson-Hot et al., 2004 [25]	IFX (retreatment with relapse or no response)	Std care (including surgery)	€63,700.82/QALY (EUR 2004)	$122,252/QALY
	IFX MTN	Std care (including surgery)	€784,057.49/QALY (EUR 2004)	$1,504,736/QALY

Kaplan et al., 2007 [26]	IFX (increasing dose)	ADA	$332,032/QALY (USD 2006)	$426,928/QALY

Lindsay et al., 2008 [27]	IFX (luminal CD)	Std care	£26,128/QALY (GBP 2006)	$60,316/QALY
	IFX (fistulizing CD)	Std care	£29,752/QALY (GBP 2006)	$68,683/QALY

Punekar et al., 2010 [28]	IFX	Std care	£14,607/QALY (GBP 2006)	$37,794/QALY

Steenholdt et al., 2014 [29]	Individualised therapy (serum IFX and IFX antibody levels using the proposed algorithm)	IFX dose intensification	Dominant	Dominant

Steenholdt et al., 2015 [30]	Individualised therapy (serum IFX and IFX antibody levels using the proposed algorithm)	IFX dose intensification	Dominant	Dominant

Tang et al., 2012 [31]	IFX	ADA	Dominant	Dominant
	IFX	Certolizumab Pegol	Dominant	Dominant
	IFX	NAT	Dominant	Dominant

Velayos et al., 2013 [32]	Testing-based strategy (IFX)	Dose escalation	Dominant	Dominant

*ADA*				

*Blackhouse et al., 2012 [21]*	*ADA*	*Std care*	*$193,305/QALY* *(CAD2011)*^*∗*^	*$198,717/QALY*
	*ADA*	*IFX*	*$451,165/QALY* *(CAD 2011)*^*∗*^	*$463,797/QALY*

Bodger et al., 2009 [22]	ADA (1 year tx)	Std care	£7,190/QALY (GBP 2006)	$18,603/QALY
	ADA (2 year tx)	Std care	£10,310/QALY (GBP 2006)	$26,676/QALY

*CADTH, 2008 [33]*	*ADA*	*Std care*	*$113,034/QALY* *(CAD 2008)*^*∗*^	*$128,067/QALY*
	*ADA*	*IFX*	*Dominant*	*Dominant*

Dretzke et al., 2011 [24]	ADA IND (severe disease)	Std care	Dominant	Dominant
	ADA MTN (severe disease)	Std care	£7,749/QALY (GBP 2011)^*∗*^	$17,888/QALY
	ADA MTN (severe disease)	ADA MTN	£4,980,000/QALY (GBP 2011)^*∗*^	$11,496,423/QALY
	ADA IND (moderate disease)	Std care	Dominant	Dominant
	ADA MTN (moderate disease)	Std care	£160,079/QALY (GBP 2011)^*∗*^	$369,545/QALY
	ADA MTN (moderate disease)	ADA IND (moderate disease)	£13,900,000/QALY (GBP 2011)^*∗*^	$32,088,410/QALY

Loftus Jr et al., 2009 [34]	ADA (severe disease)	Std care	£16,064/QALY (GBP 2006)	$38,195/QALY
	ADA (moderate-to-severe disease)	Std care	£33,731/QALY (GBP 2006)	$80,202/QALY

Yu et al., 2009 [35]	ADA MTN	IFX MTN	Dominant	Dominant

*IFX + AZA*				

Marchetti et al., 2013 [36]	IFX + AZA (top-down strategy)	Steroid (step-up strategy)	Dominant	Dominant

Saito et al., 2013 [37]	IFX + AZA	IFX	£24,917/QALY (GBP 2004)	$57,051/QALY

*Other biologics* *(GOL, NAT, VED)*				

Ananthakrishnan et al., 2012 [38]	NAT	Certolizumab Pegol	$381,678/QALY (USD 2010)	$432,011/QALY

*Immunosuppressants* *(AZA, 6MP, cyclosporine)*				

Ananthakrishnan et al., 2011 [19]	AZA	Antibiotics	Dominated	Dominated

Doherty et al., 2012 [23]	AZA/6MP	No therapy	$299,188/QALY (USD 2010)	$338,643/QALY

Priest et al., 2006 [39]	AZA	MTX	Dominant	Dominant
	AZA	No immunosuppressant therapy	Dominant	Dominant

ADA: adalimumab; AZA: azathioprine; CAD: Canadian dollar; CADTH: Canadian Agency for Drugs and Technologies in Health; CE: cost-effectiveness; EUR: Euros; GBP: Great British Pound; GMA: granulocyte-monocyte aphaeresis; GOL: golimumab; IFX: infliximab; IND: induction; MTN: maintenance; NAT: natalizumab; QALY: quality adjusted life years; Std: standard; tx: treatment; US: United States; USD: United States Dollar; VED: vedolizumab; 6MP: 6-mercaptopurine. Studies in italic are Canadian studies. A study may appear in more than one table if different treatments were analyzed. ^*∗*^Publishing year.

**Table 3:** Summary of economic evaluations in the treatment of UC.

Study, year of publication	Study treatment	Comparators	CE ratio (Cost/QALY) (Currency year)	CE ratio ($/QALY) (CAD 2016)
*IFX*				

Archer et al., 2016 [40]	IFX	Surgery	Dominated	Dominated
	IFX	ADA	Dominated	Dominated

Chaudhary and Fan, 2013 [41]	IFX	Cyclosporine	€24,277/QALY (EUR 2010)	$34,241/QALY
	IFX	Surgery	€14,639/QALY (EUR 2010)	$20,647/QALY

Hyde et al., 2009 [42]	Strategy A (IFX responders who achieved and maintained remission and mild health states)	Std care	£33,866/QALY (GBP 2009)^*∗*^	$78,180/QALY
	Strategy B (IFX responders who achieved and maintained remission)	Std care	£25,044/QALY (GBP 2009)^*∗*^	$57,814/QALY

Punekar and Hawkins, 2010 [43]	IFX	Cyclosporine	£19,545/QALY (GBP 2006)	$50,571/QALY
	IFX	Std care	£18,388/QALY (GBP 2006)	$47,578/QALY

Stawowczyk et al., 2016 [44]	IFX + std care	Std care	$106,743/QALY (USD 2015)	$135,934/QALY

*Thorlund et al., 2014 [45]*	*IFX*	*Std care*	*$65,982/QALY* *(CAD 2013)*	*$68,093/QALY*
	*IFX*	*ADA*	*Dominant*	*Dominant*

*Toor et al., 2015 [46]*	*IFX*	*Std care*	*$1,975/Remission* *(CAD 2015)*^*∗*^	*$2,038/Remission*
	*IFX*	*Std care*	*$1,311/Response* *(CAD 2015)*^*∗*^	*$1,352/Response*
	*IFX*	*GOL (100 mg)*	*$14,659/Remission* *(CAD 2015)*^*∗*^	*$15,128/Remission*
	*IFX*	*GOL*	*$4,753/Response* *(CAD 2015)*^*∗*^	*$4,905/Response*

Tsai et al., 2008 [47]	IFX MTN (responder strategy)	Std care	£27,424/QALY (GBP 2007)	$70,958/QALY
	IFX MTN (remission strategy)	Std care	£19,696/QALY (GBP 2007)	$50,962/QALY

*Ung et al., 2014 [48]*	*IFX*	*Std care*	*$79,000/QALY* *(USD 2013)*	*$85,596/QALY*

Williams et al., 2016 [49]	IFX	Cyclosporin	Dominated	Dominated

Yokomizo et al., 2016 [50]	IFX (5 mg/kg)	IFX (10 mg/kg)	$1,243,310/MH (USD 2014)	$1,366,933/MH
	IFX (5 mg/kg)	ADA	Dominated	Dominated
	IFX (5 mg/kg)	VED	Dominated	Dominated

*ADA*				

Archer et al., 2016 [40]	ADA	Surgery	Dominated	Dominated
	ADA	Std care	£50,278/QALY (GBP2015)^*∗*^	$83,804/QALY

*Thorlund et al., 2014 [45]*	*ADA*	*Std care*	*$68,722/QALY* *(CAD 2013)*	*$70,921/QALY*

*Toor et al., 2015 [46]*	*ADA*	*Std care*	*$7,430/Remission* *(CAD 2015)*^*∗*^	*$7,667/Remission*
	*ADA*	*Std care*	*$2,361/Response* *(CAD 2015)*^*∗*^	*$2,436/Response*
	*ADA*	*GOL (100 mg)*	*Dominated*	*Dominated*
	*ADA*	*GOL (100 mg)*	*Dominated*	*Dominated*

*IFX and ADA*				

*Xie et al., 2009 [51]*	*IFX (5 mg/kg) then ADA (5 mg/kg)*	*Std care*	*$358,088/QALY* *(CAD 2008)*	*$402,490/QALY*
	*IFX (5 mg/kg) then ADA (10 mg/kg)*	*Std care*	*$575,540/QALY* *(CAD 2008)*	*$646,907/QALY*

*Other biologics* *(GOL, NAT, VED)*				

Archer et al., 2016 [40]	GOL	Surgery	Dominated	Dominated

Essat et al., 2016 [52]	VED	Std care	£33,297/QALY (GBP 2016)^*∗*^	$54,283/QALY
	VED	Surgery	Dominant	Dominant
	VED (Anti-TNF naïve pt)	IFX (Anti-TNF naïve pt)	Dominant	Dominant
	VED (Anti-TNF naïve pt)	GOL (Anti-TNF naïve pt)	Dominant	Dominant
	VED (Anti-TNF naïve pt)	ADA (Anti-TNF naïve pt)	£6,634/QALY (GBP 2016)^*∗*^	$10,815/QALY
	VED (Anti-TNF naïve pt)	Std care (Anti-TNF naïve pt)	£4,862/QALY (GBP 2016)^*∗*^	$7,926/QALY
	VED (Anti-TNF naïve pt)	Surgery (Anti-TNF naïve pt)	Dominant	Dominant
	VED (Anti-TNF failure)	Std care (Anti-TNF failure)	£64,999/QALY (GBP 2016)^*∗*^	$105,966/QALY
	VED (Anti-TNF failure)	Surgery (Anti-TNF failure)	Dominant	Dominant

*Thorlund et al., 2014 [45]*	*GOL (50 mg)*	*Std care*	*$41,591/QALY* *(CAD 2013)*	*$42,921/QALY*
	*GOL (100 mg)*	*Std care*	*$42,271/QALY* *(CAD 2013)*	*$43,623/QALY*
	*GOL (50 mg)*	*IFX*	*Dominant*	*Dominant*
	*GOL (100 mg)*	*IFX*	*Dominant*	*Dominant*
	*GOL (50 mg)*	*ADA*	*Dominant*	*Dominant*
	*GOL (100 mg)*	*ADA*	*Dominant*	*Dominant*

*Toor et al., 2015 [46]*	*GOL (100 mg)*	*Std care*	*$935/Remission* *(CAD 2015)*^*∗*^	*$964/Remission*
	*GOL (100 mg)*	*Std care*	*$701/Response* *(CAD 2015)*^*∗*^	*$723/Response*
	*GOL (50 mg)*	*Std care*	*$1,048/Remission* *(CAD 2015)*^*∗*^	*$1,081/Remission*
	*GOL (50 mg)*	*GOL (100 mg)*	*$207/Remission* *(CAD 2015)*^*∗*^	*$213/Remission*
	*GOL (50 mg)*	*Std care*	*$770/Response* *(CAD 2015)*^*∗*^	*$794/Response*
	*GOL (50 mg)*	*GOL (100 mg)*	*$224/Response* *(CAD 2015)*^*∗*^	*$231/Response*

*5-ASA*				

Brereton et al., 2010 [53]	5-ASA (Mezavant XL, MMX)	5-ASA (Asacol)	£749/QALY (GBP 2010)	$2,058/QALY

Buckland and Bodger, 2008 [54]	5-ASA (High dose, Asacol)	5-ASA (Std dose, Asacol)	Dominant	Dominant

Connolly et al., 2009 [55]	5-ASA (Oral + topical)	5-ASA (Oral)	Dominant	Dominant

Connolly et al., 2009 [56]	5-ASA (2 g once daily)	5-ASA (1 g twice daily)	Dominant	Dominant

Connolly et al., 2012 [57]	5-ASA (Oral + topical)	5-ASA (Oral)	Dominant	Dominant
	5-ASA (2 g once daily)	5-ASA (1 g twice daily)	Dominant	Dominant

Connolly et al., 2014 [58]	5-ASA (2 g once daily)	5-ASA (1 g twice daily + enema)	Dominant	Dominant

Mackowiak, 2006 [59]	Oral balsalazide capsules	Oral 5-ASA specific formulation	Dominant	Dominant

Nishikawa et al., 2013 [60]	5-ASA (once daily)	5-ASA (twice daily)	$86,200/LYG (RD 2011)	$55,649/LYG

Prenzler et al., 2011 [61]	5-ASA (Mezavant XL, MMX)	5-ASA (Asacol)	Dominant	Dominant

Saini et al., 2012 [62]	SYMPT (5-ASA treatment for symptomatic disease flares only)	INFLAM (5-ASA therapy for only patients with a stool sample positive for an inflammatory marker)	$575,894/QALY (USD 2009)	$715,331/QALY
	SYMPT (5-ASA treatment for symptomatic disease flares only)	CONT (continuous 5-ASA maintenance)	Dominant	Dominant

Yen et al., 2008 [63]	MTN 5-ASA (2.4 g/day escalated and maintained at 4.8 g/day after the first flare)	No MTN 5-ASA (5-ASA 4.8 g/day given for flares)	$224,000/QALY (USD 2004)	$353,545/QALY

*Immunosuppressants* *(AZA, 6MP, cyclosporine)*				

Priest et al., 2006 [39]	AZA	MTX	Dominant	Dominant
	AZA	No immunosuppressant therapy	Dominant	Dominant

Punekar and Hawkins, 2010 [43]	Cyclosporine	Standard care	Dominant	Dominant
	Cyclosporine	Surgery	£9,032/QALY (GBP 2006)	$23,370/QALY

*Surgery*				

Archer et al., 2015 [40]	Surgery (colectomy)	Std care	Dominant	Dominant

Park et al., 2012 [64]	Surgery (early colectomy + IPAA)	Std care	$1,476,783/QALY (USD 2009)	$1,834,347/QALY

Swenson et al., 2005 [65]	Two-Stage IPAA	Three-Stage IPAA	Dominant	Dominant

*GMA*				

Panes et al., 2007 [66]	GMA	Std care	€23,898/Remission (EUR 2004)	$45,864/Remission

ADA: adalimumab; AZA: azathioprine; CAD: Canadian dollar; CE: cost-effectiveness; EUR: Euros; g: gram; GBP: Great British Pound; GMA: granulocyte-monocyte aphaeresis; GOL: golimumab; IFX: infliximab; IPAA: Ileal Pouch-Anal Anastomosis; kg: kilogram; mg: milligram; LYG: life year gained; MH: mucosal healing; MTN: maintenance; MTX: methotrexate; NAT: natalizumab; pt: patient; QALY: quality adjusted life years; RD: Real Dollar; Std: standard; US: United States; USD: United States Dollar; VED: vedolizumab; 5-ASA: 5-aminosalicylic acid; 6MP: 6-mercaptopurine. Studies in italic are Canadian studies. A study may appear in more than one table if different treatments were analyzed. ^*∗*^Publishing year.
